# Examining the factor structure of health-related quality of life in spinal cord injury using confirmatory factor analysis and multidimensional scaling

**DOI:** 10.1007/s11136-026-04354-1

**Published:** 2026-08-03

**Authors:** David S. Tulsky, Aaron J. Boulton, Pamela A. Kisala, Callie E. Tyner, Jerry Slotkin

**Affiliations:** 1 Center for Health Assessment Research and Translation, University of Delaware, Newark, DE USA; 2 Departments of Physical Therapy and Psychological & Brain Sciences, University of Delaware, Newark, DE USA

**Keywords:** Spinal cord injuries, Symptom cluster, Cluster analysis, Factor analysis

## Abstract

**Purpose:**

Spinal cord injury (SCI) is a complex medical condition and managing the multiple, co-occurring symptoms and functional and social limitations that follow SCI is a challenging task for clinical care providers. Understanding how the various post-SCI symptoms and limitations are interrelated or cluster together can inform patient care and improve clinical workflows. The purpose of this study was to determine the factor structure of the SCI-QOL measurement system and associated symptom clusters that affect health-related quality of life (HRQOL) in individuals with SCI.

**Methods:**

Participants were individuals with SCI living in the community (> 1 year after injury) recruited from 8 rehabilitation hospitals in the U.S. who completed 18 Spinal Cord Injury - Quality of Life (SCI-QOL) outcomes measures representing domains of physical, medical, social, and emotional health. Two dimension-reduction techniques, confirmatory factor analysis and multidimensional scaling, were used to analyze the data.

**Results:**

Factor analysis results suggest 7 common factors underlying the measures: Physical Symptoms, Physical Function, Negative Affect, Positive Affect, Social Health, Independence, and Pain Interference. The psychosocial factors of Negative Affect, Positive Affect, and Social Health were highly interrelated. Large correlations were also observed between Physical Function and Independence, and between Physical and Social Health. All other interrelations were small to moderate. Results from the multidimensional scaling analysis were largely consistent with those from the factor analysis.

**Conclusion:**

Study results will help inform the development of composite scores and symptom profiles in SCI, which will help researchers and clinicians better understand and manage symptoms following SCI.

**Supplementary Information:**

The online version contains supplementary material available at https://doi.org/10.1007/s11136-026-04354-1.

Understanding the interrelationships among co-occurring symptoms as well as the dynamic interplay between patient characteristics (e.g., clinical features, time since injury, co-morbidities) and clusters of symptoms has tremendous potential to transform how clinicians treat the overall symptom burden of spinal cord injury (SCI) and improve health-related quality of life (HRQOL) in the long term. Clinicians often address discrete symptoms and may fail to identify the interrelation between the symptoms and their root cause(s) [1, 2]. Recent calls for an increased focus on symptom science in clinical research [3, 4] aim to help clinicians identify related groups or “clusters” of symptoms and limitations. *Symptom clustering* is a conceptual and methodological approach defined by the analysis of two or more concurrent, interrelated symptoms, where the interaction of these symptoms may be synergistic [5–9]. Symptom clustering can personalize clinical care by revealing underlying connections between symptoms, allowing for tailoring of interventions. Further, identifying clusters *across* multiple conditions as well as those which present uniquely or differentially within specific conditions such as SCI is an aim of current symptom cluster efforts and a prerequisite for constructing holistic treatment strategies [3, 4].

Symptom clusters research has shown promise in oncology populations [10–14], and evidence is also emerging within cardiac [15–18], gastrointestinal [19], nephrological [20], musculoskeletal [21], and mental health populations [22–26]. However, symptom science remains nascent in rehabilitation settings, particularly for SCI. Some exceptions include Widerström-Noga et al.’s [27] cluster analysis of neuropathic pain phenotypes in SCI, which revealed two distinct clusters of individuals differentiated by residual spinothalamic tract function and pain catastrophizing that may necessitate different approaches to pain management. Tanadini and colleagues [28] developed data-driven approaches to stratify patients into clinically homogeneous subgroups to reduce ambiguity in the evaluation of treatment effects in clinical trials. Ehrmann et al. [29] used graphical modeling to identify four general domains of function in SCI among a large set of symptoms and functional complaints including bodily function, independence, mental health, and social participation. Within the domain of bodily function, researchers identified distinct profiles of patients differentiated primarily by impairment severity [30]. However, current work on symptom clusters in SCI is limited in its typical focus on one or two very closely related domains; further research is needed to identify related symptoms across physical, emotional, and social domains.

Tulsky and colleagues recently conducted a large study examining symptoms and profiles of participants in a sample of 755 individuals with spinal cord injury, traumatic brain injury, stroke, or major extremity injury/loss. Using confirmatory factor analysis (CFA) on scores from 23 patient-reported outcome measures (PROMs) assessing aspects of physical, emotional, and social health and 3 performance-based measures of cognition, the research team identified 10 factors (or clusters) of physical, emotional, and social issues, symptoms and limitations affecting HRQOL [31]. Latent profile analysis (LPA) was then used to identify four patient profiles that were largely condition-agnostic [32]. Each of these techniques is designed to reduce multiple variables into a more coherent and unified unit with greater reliability and clinical utility–i.e., a symptom cluster (group of interrelated symptoms) or symptom profile (group of patients with similar symptom patterns). However, results of both the factor and profile analyses identified variations in the dimensions across injury conditions that needed further work to interpret these clusters for individuals with SCI. Collinearity was observed between two emotional health factors–psychological adjustment and negative affect – suggesting that the structure of emotional well-being is nuanced and should be explored further within diagnostic groups. Additionally, variance was detected between groups on the physical function factor. Considering our prior qualitative [33] and quantitative [34, 35] work evaluating physical function in individuals with SCI, we expected a more refined model of this cluster to be needed (i.e., comprised of separate sub-constructs such as basic mobility, fine-motor function, and wheelchair mobility). Furthermore, the selection of variables applicable to four diagnostic groups resulted in the omission of key domains relevant to individuals with SCI (e.g., bowel and bladder management difficulties). Analyses in a large sample of individuals with SCI could determine whether and where these subjectively important symptoms fit in the overall structure of HRQOL after SCI and inform the most useful cluster solution for this population.

The maturation of health outcomes assessment can help advance such efforts for SCI. Several modern systems of PROMs, such as the Spinal Cord Injury-Quality of Life (SCI-QOL) [36, 37], contain brief yet psychometrically sound measures assessing physical [38–40], emotional [41–47], and social [48] symptoms and limitations and are readily accessible for use in clinical care. Input from individuals with SCI (*n* = 136) and SCI clinicians (*n* = 76) directly informed SCI-QOL development [33, 49]. All item banks are available as fixed-length forms or computer adaptive tests (CATs). The SCI-QOL includes measures of SCI-specific issues (e.g., bladder management, wheelchair mobility) that contribute to HRQOL after injury, lend themselves to identifying clusters of symptoms in individuals with SCI, and represent a step forward in building personalized medicine approaches for rehabilitation medicine.

The purpose of this study was to examine the factor structure of the SCI-QOL HRQOL measures and how symptoms and limitations cluster in individuals with SCI. We build on the results found in the cross-disability sample [31] with measures that are more unique to SCI. We also sought to test the robustness of the core clusters [31] in a larger, SCI-only sample and, because there is not yet consensus on the best analytic method to identify and explore clusters in context [3], across analytic techniques.

## Methods

### Overview and study participants

Data for the primary analyses in this study were obtained as part of a larger validation study of the SCI-QOL measurement system. Participants were 523 individuals with medically documented SCI recruited from 8 Spinal Cord Injury Model System sites in the US. Eligible participants were ≥ 18 years old, had a confirmed diagnosis of traumatic SCI, were at least 1 year post injury, and had the ability to read English. All participants provided informed consent, in accordance with the Institutional Review Board (IRB)-approved procedures. We also used data from 190 individuals with medically confirmed SCI who participated in the Tulsky et al. [31] cross-disability study in order to cross-validate the SCI-specific symptom cluster model (described below). The same measures, eligibility criteria, and study procedures were used for this sample (see Tulsky et al. [31] for additional details).

### Measures

*SCI-QOL*. The SCI-QOL contains 19 item response theory-calibrated item banks and 3 fixed-length short forms [37] that assess HRQOL in individuals with SCI. A subset of these measures is focused on physical function in SCI and known alternatively as the Spinal Cord Injury-Function Index [34, 35]. For this study, 18 of the 19 SCI-QOL item banks were analyzed, covering aspects of physical, emotional, and social HRQOL (Ambulation was excluded due to less than a third of the sample reporting the ability to ambulate). A list of the measures administered is shown in Table 1. Responses to all SCI-QOL items are provided on 4- or 5-category Likert response scales. All measures were scored on a T metric (M = 50, SD = 10) [36, 37]. More information on the SCI-QOL is available in the supplementary material.

**Table 1:** CFA models

SCI-QOL Measure	M1	M2	M3	M4	M5	M6	M7	M8	M9^a^
Bowel Management Difficulties	GEN	PHY	PHY	PSX	PSX	PSX	PSX	PSX	PSX
Bladder Management Difficulties	GEN	PHY	PHY	PSX	PSX	PSX	PSX	PSX	PSX
Basic Mobility	GEN	PHY	PHY	PFX	PFX	PFX	PFX	PFX	PFX
Self Care	GEN	PHY	PHY	PFX	PFX	PFX	PFX	PFX	PFX
Fine Motor Function	GEN	PHY	PHY	PFX	PFX	PFX	PFX	PFX	PFX
Wheelchair Mobility	GEN	PHY	PHY	PFX	PFX	PFX	PFX	PFX	PFX
Anxiety	GEN	PSY-SOC	PSY	PSY	PSY	NEG	NEG	NEG	NEG
Depression	GEN	PSY-SOC	PSY	PSY	PSY	NEG	NEG	NEG	NEG
Psychological Trauma	GEN	PSY-SOC	PSY	PSY	PSY	NEG	NEG	NEG	NEG
Grief & Loss	GEN	PSY-SOC	PSY	PSY	PSY	NEG	NEG	NEG	ADJ
Self-Esteem	GEN	PSY-SOC	PSY	PSY	PSY	NEG	NEG	NEG	ADJ
Stigma	GEN	PSY-SOC	PSY	PSY	PSY	NEG	NEG	NEG	ADJ
Pain Interference	GEN	PSY-SOC	PSY	PSY	PSY	NEG	PSX	PAIN	PAIN
Positive Affect & Well-Being	GEN	PSY-SOC	PSY	PSY	PSY	POS	POS	POS	POS
Resilience	GEN	PSY-SOC	PSY	PSY	PSY	POS	POS	POS	POS
Ability to Participate in SRA	GEN	PSY-SOC	SOC	SOC	SOC	SOC	SOC	SOC	SOC
Satisfaction with SRA	GEN	PSY-SOC	SOC	SOC	SOC	SOC	SOC	SOC	SOC
Independence	GEN	PSY-SOC	SOC	SOC	IND	IND	IND	IND	IND

The first factor, PSX, is a new, SCI-specific factor. All other factors are represented in the global model, although constituent variables in some cases differed. For PFX, a completely different set of SCI-specific variables were used

^a^Model which most closely mirrors the global model in Tulsky et al. [50]

*Diff* difficulties, *SRA* social roles and activities, *GEN* general health, *PHY *  physical health, *PSX*  physical symptoms, *PFX* physical function, *PSY*  psychological health, *SOC* social health, *PSY-SOC*  psychosocial health, *IND*  independence, *NEG*  negative affect, *POS* positive affect, *PAIN* pain interference, *ADJ*  psychological adjustment

## Data collection procedures

For the primary analysis sample, SCI-QOL CATs were administered in a standardized interview format in person or by phone. Trained interviewers read each question and its response choices and recorded participants’ responses directly into the Assessment Center™ platform [51]. All SCI-related information was confirmed through medical record review. Similar procedures were used for the cross-validation sample study, although all interviews were conducted by phone and fixed-length forms were administered instead of CATs.

## CFA model specification

Our research team specified 9 models, shown in Table 1, based on our previous work, including the global model reported in Tulsky et al. [31], as well as results from qualitative data analysis [33, 49]. Detail on the theoretical backing for competing CFA models is included in the supplementary material. Models increased in complexity, with the last model (Model 9) most closely mirroring the global solution [31], although differences emerged: (a) the Physical Function domain (“cluster”), which was comprised of measures of lower extremity (mobility) and upper extremity (self-care) function in Tulsky et al. [31], was instead represented by four SCI-FI item banks: Basic Mobility, Fine Motor Function, Self-Care, and Wheelchair Mobility; (b) SCI-QOL measures of Bladder and Bowel Management Difficulties and Psychological Trauma, which were not part of the global model, were included; (c) some domains/more universal symptoms not included in the SCI-QOL were not represented in the CFA models, including Cognition, Fatigue/Sleep Impairment, and Economic QOL, as well as measures of Anger, Pain Intensity, and Nociceptive Pain. Of interest was whether Model 9, which most closely mirrored the global model [31], would provide the best cluster representation despite the slightly different included variables. Other questions captured by the model comparisons include whether the bowel and bladder variables would cluster with other physical symptoms, and whether pain interference and independence would continue to load as separate factors or possibly cluster with other symptoms – for example, whether pain interference would cluster with physical or mental health symptoms [52].

### Data analysis

Data were analyzed using two data reduction techniques – CFA and multidimensional scaling (MDS).

*Confirmatory Factor Analysis (CFA).* A total of 9 CFA models were specified, estimated, and compared (Table 1) to select an optimal representation of the data. To estimate each model, the *lavaan* package (version 0.6–19) [53] was used within R (version 4.2.2) [54]. The SCI-QOL Self-Esteem measure [47] was reverse coded to enhance interpretability. Missing data were handled via full information maximum likelihood estimation, which provides comparable performance to multiple imputation and uses all available information in the dataset. The specific estimator used (“MLR” in Mplus/lavaan parlance) provided robust standard errors for model parameters and a robust chi-square test of model fit. Each model was examined with regard to parameter estimate interpretability and fit to the data; specific fit indices (and fit thresholds) include the Comparative Fit Index (CFI [55]; acceptable fit > 0.90, excellent fit > 0.95), Tucker-Lewis Index (TLI [56]; acceptable fit > 0.90, excellent fit > 0.95), Root Mean Square Error of Approximation (RMSEA [57]; acceptable fit < 0.08, excellent fit < 0.06), and Standardized Root Mean Square Residual (SRMR [58]; acceptable fit < 0.06). Additionally, the Bayesian Information Criterion (BIC) was used to compare models, with the lowest value indicative of optimal comparative fit. After comparing results, a final model was chosen. This model was then fit in the cross-validation sample; fit index values and parameter estimates derived from the primary analysis and the cross-validation fit were then compared.

*Multidimensional Scaling (MDS).* MDS is a flexible set of methods used for non-parametric dimension reduction [59, 60]. This additional technique was applied because (a) it offered a non-parametric comparison to the CFA results, (b) it provided a visualization of how the SCI-QOL measures cluster together graphically, and (c) related to (b), it allowed a complementary examination of factors indicated by only one or two SCI-QOL measures, which are locally under-identified and thus require an assumption of no measurement error for one-indicator factors or possibly restrictive assumptions for two-indicator factors (e.g., equality constraints on factor loadings).

The analyses carried out for this project relied on modern improvements to the approach, specifically the *stress majorization* procedure as implemented in the *smacof* R package (version 2.1-0) [61]; this is an iterative estimation routine that attempts to minimize the distance between input dissimilarities (non-parametric distances between each variable pair) and those predicted by a low dimensional configuration. The sample dissimilarity matrix was computed using Euclidean distances between variable pairs. Missing data were handled in a pairwise deletion fashion. The first step in MDS is to select the number of dimensions required for data reduction–note that this does not have the same meaning as the number of factors in CFA – that provides the best tradeoff between model fit and parsimony. This step was carried out by inspecting stress values for MDS configurations ranging from 1 to 7 dimensions. Stress values were plotted and inspected visually to identify the point(s) at which improvement in fit becomes negligible with additional dimensions, referred to as the “elbow” of the plot. Dimensions represented by the elbow were then further explored. Because MDS configurations can be sensitive to parameter starting values, 100 sets of random start values were generated for each candidate dimension and used for analysis, resulting in 100 MDS configurations per dimension. Following Mair, Groenen, and de Leeuw [62], configurations for each dimension examined were represented in a 2-dimensional space using Procrustean fitting techniques, and candidate configurations were examined for interpretability. Final MDS configurations were chosen from clusters featuring configurations that could be meaningfully interpreted and exhibited the smallest stress values possible. The final configurations were further examined for interpretability and compared. All analyses were conducted in R (version 3.6.3) [54].

## Results

### Participants

Demographic and injury characteristics for the primary analysis and cross-validation samples are shown in Table 2. For the primary analysis sample, the average age was 43.1 years (SD = 15.2) and 78% were male. Average time since injury was 4.6 years (SD = 6.7). Fewer participants were diagnosed with paraplegia (*n* = 231, 44.2%) than tetraplegia (*n* = 290, 55.4%). The cross-validation sample exhibited distributions similar to the primary analysis sample for all characteristics shown in Table 2.

**Table 2:** Participant demographic and injury characteristics

	Primary analysis sample (*n* = 523)		Cross-validation sample (*n* = 190)	
	M	(SD)	M	(SD)
Age	43.1	(15.2)	50.0	(15.7)
Time Since Injury (Years)	4.6	(6.7)	14.8	(12.0)
	*N*	(%)	*N*	(%)
Gender				
Female	115	(22)	41	(20.3)
Male	408	(78)	161	(79.7)
Race				
White	401	(77)	133	(70)
Black	77	(15)	39	(21)
Asian	8	(2)	5	(3)
American Indian / Alaskan Native	3	(1)	0	(0.0)
Native Hawaiian / Pacific Islander	2	(0)	0	(0.0)
Multiracial	17	(3)	5	(3)
Other	9	(2)	8	(4)
Not Provided	6	(1)	0	(0)
Ethnicity				
Hispanic	42	(8)	30	(16)
Non-Hispanic	463	(89)	160	(84)
Not Provided	18	(3)	0	(0.0)
Injury Diagnosis				
Paraplegia	231	(44)	80	(42)
Tetraplegia	290	(55)	89	(47)
Not Provided	2	(0)	21	(11)
Injury Completeness				
Complete	228	(44)	80	(42)
Incomplete	277	(53)	88	(46)
Not Provided	18	(3)	22	(12)

## CFA results

Missingness was generally low for the 18 SCI-QOL measures; missing data rates were less than 1% for all measures except Independence (16.6%) and Wheelchair Mobility (18.5%) Both cases were due to structural missingness; for Wheelchair Mobility, items were only administered to people who reported using a manual or power wheelchair, and for Independence, a random subset of participants did not receive this measure. Fit information for all models estimated are shown in Table 3. The best-fitting solutions were found for Models 6, 8, and 9 according to the BIC, although alternative fit indices were similar across the three models. Although Model 9 exhibited the closest fit among all models, the correlation between the Negative Affect and Psychological Adjustment factors was 0.96 and thus could not be meaningfully distinguished. As a result, Model 9 was not considered further. For Models 6 and 8, both solutions met acceptable or excellent fit thresholds and contained the factors Physical Symptoms, Physical Function, Negative Affect, Positive Affect, Social Health, and Independence. In Model 6, Pain Interference loaded onto the Negative Affect factor (factor loading = 0.54), whereas in Model 8, Pain Interference constituted a single-indicator factor that was correlated with all other latent dimensions. Notably, both models provided a closer fit to the data than when Pain Interference was specified to load onto the Physical Symptoms factor (Model 7). Both models suggest a moderate correlation between Pain Interference and the other Negative Affect indicators. In the following paragraph, we report estimates from Model 8, which we considered to have the best empirical support.

**Table 3:** CFA model fit comparison

Model	χ^2^	df	*p*	CFI	TLI	RMSEA	RMSEA 90% C.I.	SRMR	BIC
1	2735.7	135	< 0.01	0.631	0.581	0.211	[0.204, 0.218]	0.160	103977.2
2	1121.5	134	< 0.01	0.858	0.838	0.131	[0.124, 0.138]	0.131	102162.4
3	815.2	132	< 0.01	0.903	0.888	0.109	[0.102, 0.117]	0.106	101821.3
4	646.6	129	< 0.01	0.925	0.911	0.097	[0.090, 0.105]	0.061	101649.9
5	474.8	126	< 0.01	0.952	0.942	0.079	[0.071, 0.086]	0.038	101478.6
6	379.2	121	< 0.01	0.965	0.955	0.069	[0.061, 0.077]	0.036	101399.4
7	437.1	121	< 0.01	0.957	0.946	0.076	[0.068, 0.084]	0.043	101461.8
8	355.5	116	< 0.01	0.967	0.956	0.068	[0.060, 0.076]	0.033	101404.6
9	311.1	109	< 0.01	0.972	0.961	0.065	[0.056, 0.073]	0.028	101396.0

**Fig. 1:**
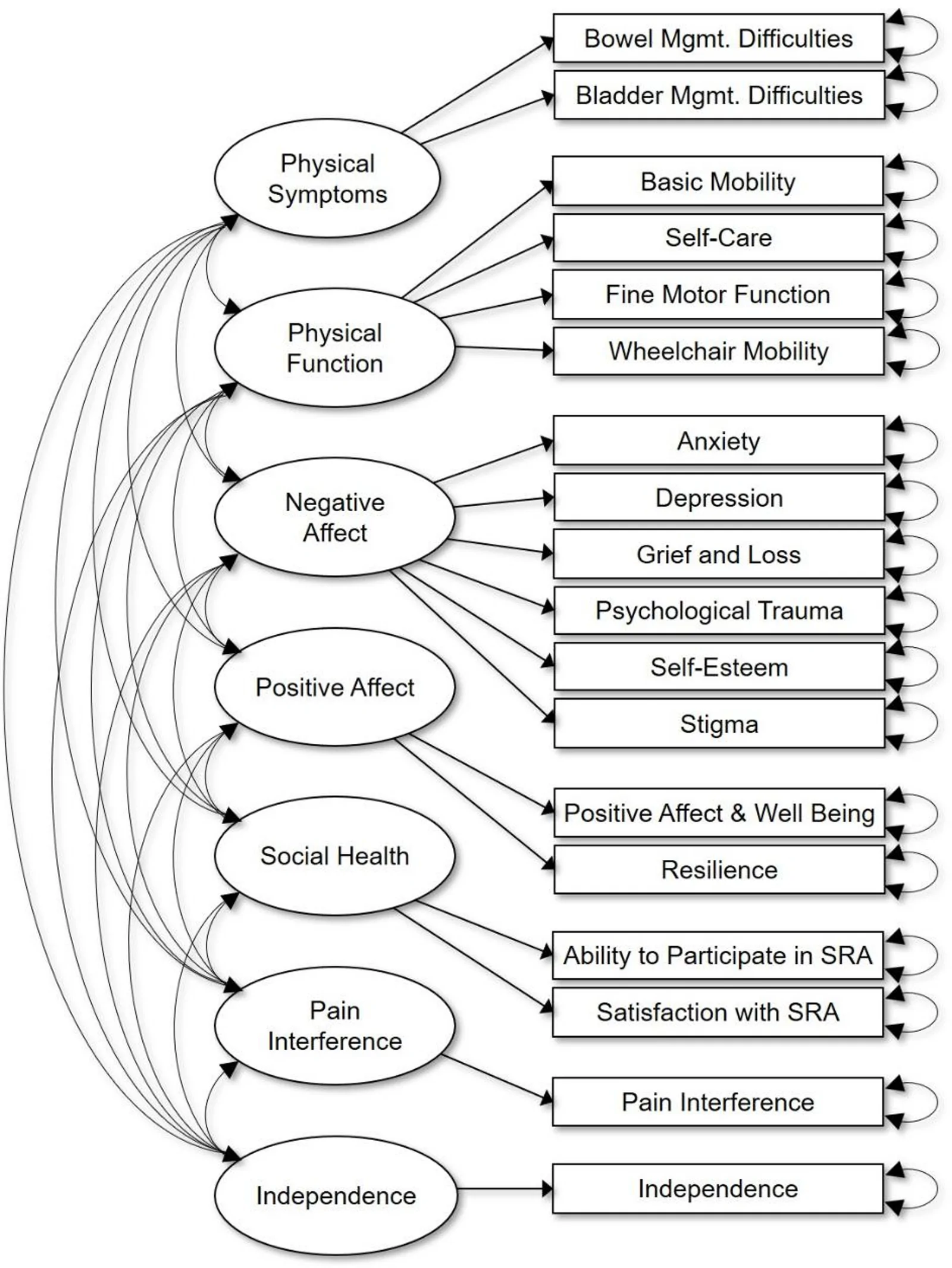
Path diagram for model 8

Standardized parameter estimates for Model 8 are shown in Table 4 (factor loadings and residual variances) and Table 5 (factor correlations). A path diagram of Model 8 is shown in Fig. 1. Of note, the Physical Symptoms factor was only moderately correlated with all other factors, including Physical Function. The three psychosocial factors – Negative Affect, Positive Affect, and Social Health – were strongly correlated, although a model in which these factors (along with Independence) were merged (Model 2) did not fit the data as well as Model 8. A partial second-order version of Model 8 in which the three psychosocial factors were specified as first-order indicators of an Emotional Health factor fit about as well as Model 8, χ2 (124) = 484.9, *p* < .01, RMSEA = 0.081 [0.073, 0.089], CFI = 0.950, TLI = 0.938, SRMR = 0.052. See the Discussion section below for consideration and comparison of the first- and partial second-order model.

Likewise, despite their strong correlation, the model in which the single-indicator factor Independence was merged with the Social Health factor (Model 4) was not optimal. A model in which Independence had a dual loading on both Physical Function and Social Health likewise was not the best fitting solution (results not shown). Finally, as further support for Model 8, we conducted tests of measurement invariance across individuals with paraplegia and tetraplegia. We discuss these tests and accompanying results (i.e., all factors were invariant except for Physical Function) in the Supplementary Material.

**Table 4:** Standardized parameter estimates for model 8

SCI-QOL measure	Factor loadings		Residual variances	
	Std. Est.	SE	Std. Est.	SE
*Physical Symptoms*				
Bowel Management Difficulties	0.80	0.07	0.36	0.11
Bladder Management Difficulties	0.57	0.06	0.68	0.06
*Physical Function*				
Basic Mobility	0.88	0.01	0.22	0.02
Self Care	0.96	0.01	0.08	0.01
Fine Motor Function	0.90	0.01	0.19	0.02
Wheelchair Mobility	0.91	0.01	0.18	0.02
*Negative Affect*				
Anxiety	0.84	0.02	0.30	0.03
Depression	0.91	0.01	0.17	0.02
Grief and Loss	0.88	0.01	0.22	0.02
Psychological Trauma	0.82	0.02	0.33	0.03
Self-Esteem ^b^	0.93	0.01	0.14	0.02
Stigma	0.76	0.02	0.42	0.03
*Positive Affect*				
Positive Affect and Well-Being	0.91	0.02	0.18	0.03
Resilience	0.90	0.01	0.19	0.02
*Social Health*				
Ability to Participate in Social Roles and Activities (SRA)	0.81	0.02	0.35	0.04
Satisfaction with SRA	0.88	0.02	0.23	0.03
*Pain Interference*				
Pain Interference	1.00^a^	n/a	0.00	n/a
*Independence*				
Independence	1.00^a^	n/a	0.00	n/a

All factor variances were fixed to 1.0 for model identification

*SRA*  social roles and activities

^b^ Reverse-coded to enhance interpretability

**Table 5:** Factor Correlations

Physical symptoms	Physical symptoms	Physical function	Negative affect	Positive affect	Social health	Independence	Pain interference
Physical function	− 0.21						
Negative affect	0.49	− 0.29					
Positive affect	− 0.41	0.31	− 0.91				
Social health	− 0.41	0.52	− 0.84	0.84			
Independence	− 0.36	0.76	− 0.52	0.50	0.74		
Pain interference	0.31	− 0.04	0.53	− 0.46	− 0.49	− 0.25	

Results from the cross-validation sample were highly similar to those in the primary analysis sample. Similar model fit values were obtained, which likewise met conventional thresholds of acceptability: χ^2^ (109) = 206.675, CFI = 0.965, TLI = 0.954, RMSEA = 0.068 [90% CI = 0.053, 0.083], SRMR = 0.041. Parameter estimates were also highly similar across samples. Standardized factor loadings differed between samples on average by 0.048 (SD = 0.054). Correlations differed between samples on average by.061 (SD = 0.073).

## MDS results

**Fig. 2:**
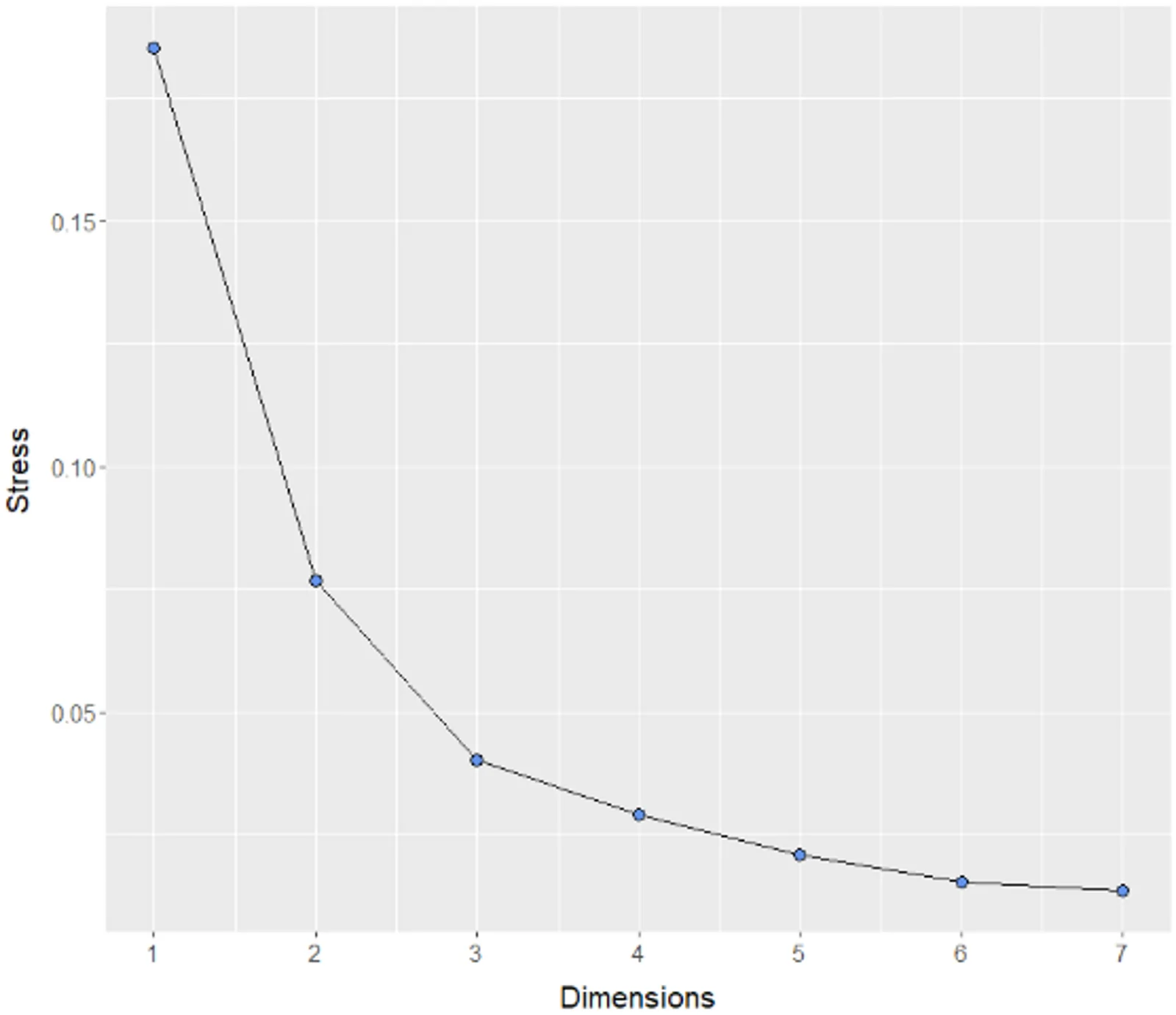
Stress plot for MDS analysis

Stress values are shown for dimensions ranging from 1 to 7 in Fig. 2. A clear elbow emerges at three dimensions; thus, 2- and 3-dimensional solutions were further explored. One hundred random start configurations for 2- and 3-dimensional solutions (2-D and 3-D) were computed and the solution with the smallest stress value was chosen. It was determined that a 3-D solution (stress = 0.046) provided a more interpretable solution compared to the 2-D solution; thus the 2-D solution is not considered further. A permutation test showed that the 3-D stress value for the final solution was significantly below most values from a null distribution based on randomly permuted data – that is, the 3-D configurations exhibited good fit to the data.

**Fig. 3:**
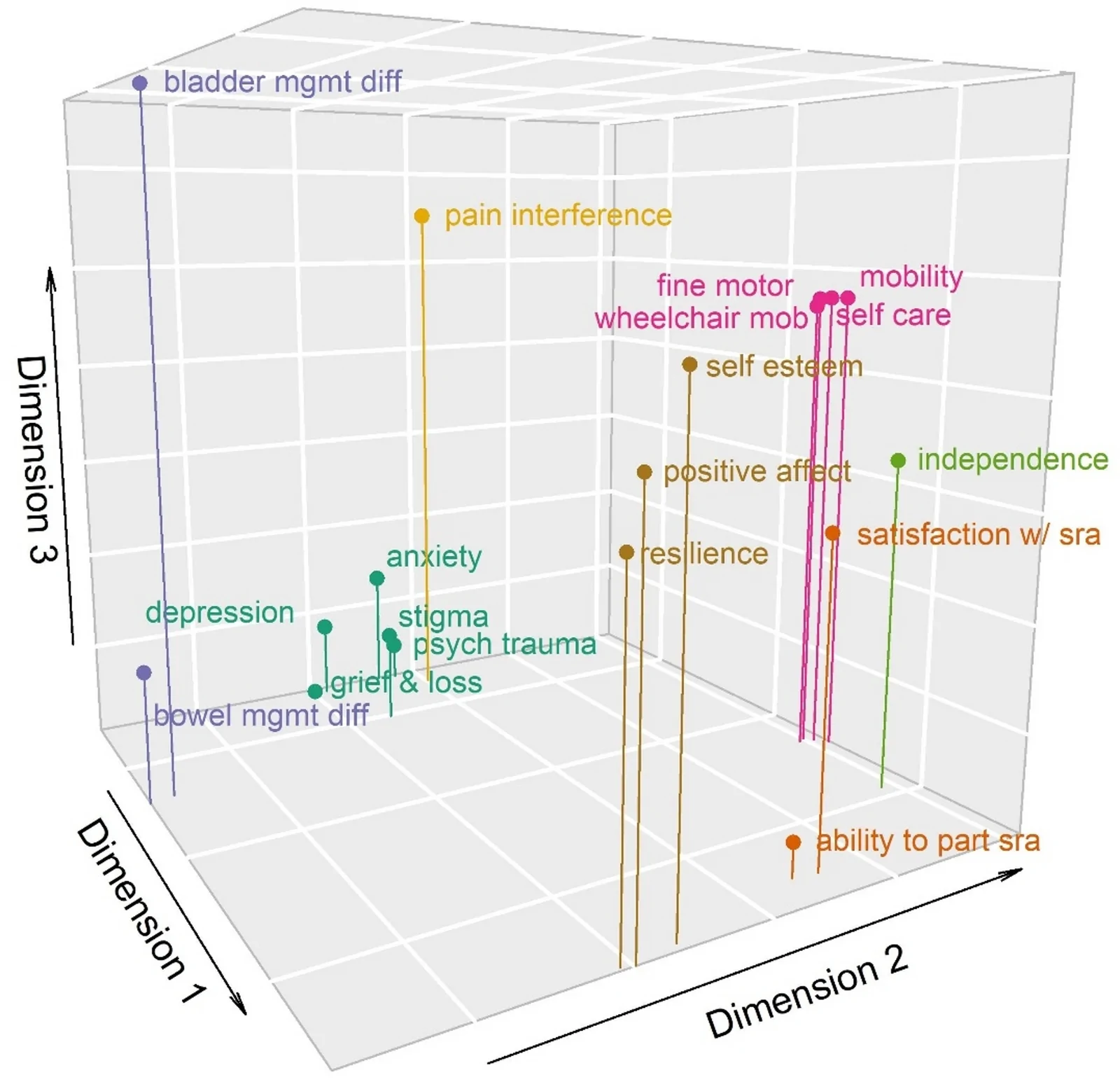
Three-dimensional representation of final MDS solution

The final 3-D configuration chosen is shown in Fig. 3. Dimensions in MDS solutions are not generally interpretable in MDS; rather it is the relative distances between variables that capture how variables cluster together. However, in Fig. 3, it appears that the first dimension may be interpreted as “valence,” given the strong polarity between the variables that could be categorized as positive vs. negative valence. In the figure, seven clusters emerged: A Physical Function cluster (Basic Mobility, Self-Care, Fine Motor Function, Wheelchair Mobility), a Social Health cluster (Ability to Participate in SRA, Satisfaction w/SRA), a Positive Affect cluster (Positive Affect & Well-Being, Resilience, Self-Esteem), a Negative Affect cluster (Anxiety, Depression, Grief & Loss, Stigma, Psychological Trauma), a Pain cluster (Pain Interference), an Independence cluster (Independence), and a Physical Symptoms cluster (Bladder Management Difficulties, Bowel Management Difficulties). The clusters varied with respect to homogeneity (i.e., how closely variables within the same cluster were related). For instance, the Negative Affect, Positive Affect, and Physical Function clusters exhibited high within-cluster homogeneity, whereas the Social Health and Physical Symptoms clusters did not. Indeed, a case could be made for further separating the Physical Symptoms cluster into the two constituent symptoms, although these variables were well-differentiated from the other clusters along the second dimension. In comparison to the CFA solution, only minor differences emerged. Notably, Self-Esteem belonged to the Positive Affect cluster in the MDS solution, although this may have been an artifact due to the variable not being inverted in the MDS analysis. Therefore, the MDS solution largely replicated the CFA results.

## Discussion

The health consequences of a traumatic SCI are often numerous and can affect multiple aspects of health. Therefore, it is imperative for clinicians and researchers to understand the relations among all pertinent symptoms and/or impairments that can arise following SCI. Medical complaints that tend to co-occur may be more efficiently managed if considered through a symptom cluster lens, especially if they arise due to a singular underlying cause, or by a central “driving” symptom [63]. As a first step, in this study we examined the underlying latent structure of the SCI-QOL measurement system, which presents a comprehensive clinical picture of HRQOL following traumatic SCI. Two related but distinct analytic approaches-CFA and MDS–were applied to data from a large cohort of respondents living in the community with SCI who completed 18 SCI-QOL measures. Results of the CFA suggested the SCI-QOL measures were underpinned by 7 common factors: Physical Symptoms, Physical Function, Pain Interference, Negative Affect, Positive Affect, Social Health, and Independence. Correlations among the three psychosocial factors were large, as were correlations between Physical Function and Independence and Physical Function and Social Health. Despite these strong relations, the model with these six factors as distinct dimensions was considered optimal. Results from the MDS analysis–a nonparametric and graphical approach to dimension reduction–were largely consistent with those observed in the CFA. Furthermore, a partial second-order version of Model 8 with the 3 psychosocial factors (Negative Affect, Positive Affect, and Social Health) subsumed into a higher-order Emotional Health factor fit about as well; Model 8 is still supported due to conceptual differentiation of Emotional Health subdomains and is our preferred model. However, the second-order model has psychometric support as indicated by fit indices as an alternative structure.

The identified 7-factor solution, with each factor representing a “cluster” of symptoms, is consistent with our team’s prior work on cross-disability (stroke, SCI, traumatic brain injury [TBI], limb trauma/loss) clusters of symptoms. The replication of a core set of factors–including Positive Affect, Negative Affect, Physical Symptoms, Physical Function, and Social Participation – across multiple studies, samples, and even populations suggests a robustness to the structure of HRQOL for individuals with disabilities. Of course, SCI-specific differences also emerged. The Physical Symptoms factor is comprised solely of the SCI-QOL Bladder Management Difficulties and Bowel Management Difficulties scores, and measures comprising the construct of Physical Function are more nuanced for SCI, with the multiple different subcomponents of basic mobility, self-care, fine motor function, and wheelchair mobility contributing to this cluster. The Psychological Adjustment factor, identified in the cross-disability model [31] as well as within TBI (wherein it was referred to as “Sense of Self”) [64], did not emerge due to its highly collinear relation to the Negative Affect cluster. However, there are slight differences in the variables that were included (e.g., a measure of Anger was not included, whereas a measure of Psychological Trauma was) and it is unclear if these variables account for the differences in results or if it is a true difference between conditions in the convergence of these two clusters. Because four different diagnostic groups were included in the cross-disability cluster model, it is possible that the distinguishability between these clusters arising from the other three conditions outweighed those observed in SCI.

Results suggest that the field would benefit from a way to measure across these robust clusters, and then if a given cluster is flagged, for clinicians to be able to examine individual symptom/measures scores more closely. In the field of psychological measurement, composite or “index” scores are routinely used for this purpose; development of composite scores for SCI-QOL measures will be an important future direction for this work.

### Limitations

There were several limitations to the current study. Despite the large breadth of the SCI-QOL system [37], there were symptoms omitted from the analyses. These omissions were either due to low endorsement rates in the sample obtained – for instance, Ambulation – or those that are not currently measured by the SCI-QOL system (e.g., Sleep Disturbance). Future work should consider whether additional symptoms also form or contribute to clusters of relevance to individuals with SCI. Second, only a single measure of pain was included in this study. The SCI-QOL Pain Interference measure addresses the extent to which chronic (undifferentiated) pain interferes with daily activities. Given the potential important role of pain in SCI, additional measures that address pain severity, subtypes, or coping mechanisms may also prove useful. Finally, there is not yet consensus regarding the optimal analytic method to identify symptom clusters. Although two related but distinct methods were used in the current study, it is possible that other approaches would have resulted in different results. Thus, additional research evaluating the robustness of the structures reported here is warranted.

## Conclusion

Seven clusters of HRQOL-affecting symptoms and limitations were identified using CFA and replicated using MDS variable reduction techniques. Each cluster represents a potential target for clinical intervention and can inform future development of SCI-QOL composite scores and symptom profiles with a goal of improving understanding and treatment of the array of co-occurring symptoms following SCI.

## Supplementary Information

Below is the link to the electronic supplementary material.

Supplementary Material 1

## Data Availability

Data sharing is not applicable to this article as no new data were created or analyzed in this study.
