# Supplementary material for: Examining the factor structure of health-related quality of life in spinal cord injury using confirmatory factor analysis and multidimensional scaling
Source: Qual Life Res. 2026 Aug 3;35(9):255. doi: 10.1007/s11136-026-04354-1 (PMC13433718; doi:10.1007/s11136-026-04354-1)
Supplement: Supplementary file 1 [file 11136_2026_4354_MOESM1_ESM.docx]

Supplementary Material

**Title:** Examining the Factor Structure of Health-Related Quality of Life in Spinal Cord Injury Using Confirmatory Factor Analysis and Multidimensional Scaling

***Journal:*** *Quality of Life Research*

**Authors:**

David S. Tulsky, Ph.D., University of Delaware, dtulsky@udel.edu

Aaron J. Boulton, Ph.D.

Pamela A. Kisala, M.A.

Callie E. Tyner, Ph.D.

Jerry Slotkin, Ph.D.

This supplement provides additional detail regarding the measures, models, and findings reported in the main article, including:

- Information on the development and contents of the SCI-QOL measurement system.
- Details on the theoretical backing for the selection of the alternate CFA models outlined in the main paper.
- Descriptive statistics (means, SDs, correlations) for the 18 SCI-QOL measures included in the CFA and MDS analyses in the main paper (presented as Tables S1 and S2).
- Details and results regarding measurement invariance analyses of the selected CFA model across participants with paraplegia or tetraplegia that are referenced in the main paper.

The Spinal Cord Injury – Quality of Life (SCI-QOL) Measurement System.

The SCI-QOL is a condition-specific extension of the Patient Reported Outcomes Measurement Information System (PROMIS^®^) and Neurological Quality of Life (Neuro-QOL^TM^) that has been optimized for individuals with SCI. The SCI-QOL includes 19 item response theory-calibrated item banks and 3 fixed-length scales assessing physical, emotional, and social aspects of health-related QOL [1], with domains and items selected and developed using a systematic mixed-methods approach that centers on input from individuals with SCI and SCI clinicians [2; 3]. Domains include Positive Affect & Well-being [4], Depression [5], Anxiety [6], Stigma [7], Resilience [8], Grief/Loss [9], Self-Esteem [10], Psychological Trauma, Pressure Ulcers [11], Bladder Management Difficulties, Bladder Complications [12], Bowel Management Difficulties [12], Pain Interference [13], Pain Behavior, Ability to Participate in Social Roles & Activities, Satisfaction with Social Roles & Activities [14], Independence, Basic Mobility, Ambulation, Fine Motor, Self-Care, and Wheelchair Mobility [15]. This work has been described in depth in a special issue of the Journal of Spinal Cord Medicine. There are overview[15] and methodology[1] manuscripts which would be helpful for novel users of the system, as well as domain-specific manuscripts with additional information on the conceptual development and psychometric properties of individual item banks. The physical function item banks are also known as the Spinal Cord Injury-Functional Index[16-18] and were similarly developed with iterative input from individuals with SCI and SCI clinicians.[19] All SCI-QOL measures were developed in accordance with the PROMIS Instrument Development and Psychometric Evaluation Scientific Standards.[20]

Theoretical Background of CFA Model Selection.

This team’s previous research demonstrated a complex factor structure underlying quality of life in individuals with disabilities (see Tulsky *et al*. [21] for a model in a mixed disability sample and *Sherer et al.* [22] for a model in a large sample of individuals with traumatic brain injury). These previous studies have supported evidence that a model with factors defined by Physical Symptoms, Physical Function, Positive Affect, Negative Affect, Social Function, Independence, and Psychological Adjustment. Hence, we started analyzing our data utilizing this structure as a base. But at the core of our model development, we hypothesized that this multifactorial structure would be supported by the fit indices when parallel models were tested in an SCI population.

The current investigation also included measures of SCI-specific constructs like wheelchair mobility, bowel management, and bladder management that were not included in our prior research. We also envisioned pain to be a unique construct in many individuals with SCI who experience pain *in the absence of* other sensations. This appeared to be categorically different from other physical symptoms such as bowel and bladder functioning.

At the same time, we developed models with more simplistic structures. The simplest (most parsimonious) model would be model 1, a single factor of general HRQOL. Then we move to model 2, which splits overall HRQOL into a physical vs. a psychosocial factor. Model three represents the common tripartite structure first advanced by the World Health Organization that emphasizes a structure of physical, emotional, and social functioning. The specification and examination of model fit for these simple models allowed us to ensure that our final model wasn’t needlessly complex, while still allowing us to test alternate hypotheses.

We also developed some competing models with different placement of SCI-specific issues (e.g., bowel and bladder management, pain, and subcomponents of physical functioning such as basic mobility, wheelchair mobility, and fine motor functioning). In Models 4 and 5, we were able to separate physical symptoms from physical functioning. We also brought out the construct of Independence that was separate from social functioning. As we developed more complicated models, we introduced separation between negative and positive emotional functioning (models 6 and 7). We considered models with pain loading with negative emotional functioning (model 6), physical symptoms (model 7), or as a separate factor by itself (Model 8). Finally, we modified Model 8 to separate negative emotions such as grief/loss, self-esteem, and stigma into a separate factor related to psychological adjustment, representing the structure we found in our previous studies. This final model is represented in Model 9.

Table S1. *Descriptive Statistics for SCI-QOL Measures*

|  | *n* | M | SD |
| --- | --- | --- | --- |
| Ability to Participate in SRA | 521 | 48.9 | 9.6 |
| Anxiety | 521 | 50.8 | 9.1 |
| Bladder Management Difficulty | 521 | 51.5 | 9.6 |
| Bowel Management Difficulty | 520 | 48.2 | 8.1 |
| Depression | 522 | 49.2 | 8.6 |
| Fine Motor | 522 | 52.0 | 9.0 |
| Grief and Loss | 521 | 50.6 | 9.0 |
| Independence | 436 | 49.6 | 9.7 |
| Basic Mobility | 522 | 52.5 | 9.3 |
| Pain Interference | 521 | 50.1 | 9.3 |
| Positive Affect & Well-Being | 520 | 50.2 | 9.8 |
| Psychological Trauma | 521 | 50.7 | 9.3 |
| Resilience | 522 | 50.3 | 9.6 |
| Satisfaction with SRA | 520 | 50.6 | 9.6 |
| Self-Care | 522 | 52.4 | 9.3 |
| Self-Esteem | 520 | 50.5 | 9.2 |
| Stigma | 520 | 53.0 | 6.4 |
| Wheelchair Mobility | 426 | 51.5 | 9.1 |
| Ability to Participate in SRA | 521 | 48.9 | 9.6 |
| Anxiety | 521 | 50.8 | 9.1 |
| Bladder Management Difficulty | 521 | 51.5 | 9.6 |
| Bowel Management Difficulty | 520 | 48.2 | 8.1 |
| Depression | 522 | 49.2 | 8.6 |
| Fine Motor | 522 | 52.0 | 9.0 |
| Grief and Loss | 521 | 50.6 | 9.0 |

*Note*. Several SCI-QOL measures are linked to the PROMIS or Neuro-QoL systems (e.g., PROMIS Depression) and scores are typically calculated in reference to the metric of the source measure (e.g., general U.S. population for PROMIS Depression). For the present study, however, all SCI-QOL measures were scored on a T metric in reference to the SCI population using item parameters estimated in the original SCI calibration sample. See Tulsky *et al.* [1; 15] for further detail.

Table S2. *Correlations Between SCI-QOL Measures*

|  | Ability to Participate in SRA | Anxiety | Bladder Management Difficulty | Bowel Management Difficulty | Depression | Fine Motor | Grief and Loss | Independence | Basic Mobility | Pain Interference | Positive Affect & Well-Being | Psychological Trauma | Resilience | Satisfaction with SRA | Self-Care | Self-Esteem | Stigma | Wheelchair Mobility |
| --- | --- | --- | --- | --- | --- | --- | --- | --- | --- | --- | --- | --- | --- | --- | --- | --- | --- | --- |
| Ability to Participate in SRA | 1.00 | -0.59 | -0.15 | -0.27 | -0.63 | 0.33 | -0.63 | 0.61 | 0.38 | -0.45 | 0.61 | -0.55 | 0.62 | 0.71 | 0.38 | 0.61 | -0.48 | 0.39 |
| Anxiety | -0.59 | 1.00 | 0.26 | 0.30 | 0.77 | -0.15 | 0.71 | -0.43 | -0.19 | 0.52 | -0.65 | 0.77 | -0.68 | -0.61 | -0.18 | -0.76 | 0.62 | -0.13 |
| Bladder Management Diff. | -0.15 | 0.26 | 1.00 | 0.45 | 0.25 | -0.02 | 0.21 | -0.17 | -0.09 | 0.17 | -0.19 | 0.22 | -0.18 | -0.17 | -0.04 | -0.26 | 0.26 | 0.04 |
| Bowel Management Diff. | -0.27 | 0.30 | 0.45 | 1.00 | 0.36 | -0.17 | 0.35 | -0.31 | -0.20 | 0.25 | -0.32 | 0.29 | -0.30 | -0.32 | -0.18 | -0.36 | 0.39 | -0.12 |
| Depression | -0.63 | 0.77 | 0.25 | 0.36 | 1.00 | -0.21 | 0.79 | -0.47 | -0.27 | 0.48 | -0.79 | 0.75 | -0.78 | -0.68 | -0.25 | -0.84 | 0.67 | -0.23 |
| Fine Motor | 0.33 | -0.15 | -0.02 | -0.17 | -0.21 | 1.00 | -0.24 | 0.67 | 0.76 | -0.02 | 0.26 | -0.15 | 0.22 | 0.41 | 0.88 | 0.24 | -0.26 | 0.82 |
| Grief and Loss | -0.63 | 0.71 | 0.21 | 0.35 | 0.79 | -0.24 | 1.00 | -0.50 | -0.29 | 0.45 | -0.73 | 0.73 | -0.72 | -0.68 | -0.28 | -0.83 | 0.70 | -0.25 |
| Independence | 0.61 | -0.43 | -0.17 | -0.31 | -0.47 | 0.67 | -0.50 | 1.00 | 0.74 | -0.23 | 0.46 | -0.35 | 0.45 | 0.68 | 0.73 | 0.49 | -0.47 | 0.70 |
| Basic Mobility | 0.38 | -0.19 | -0.09 | -0.20 | -0.27 | 0.76 | -0.29 | 0.74 | 1.00 | -0.05 | 0.27 | -0.19 | 0.26 | 0.43 | 0.84 | 0.27 | -0.29 | 0.85 |
| Pain Interference | -0.45 | 0.52 | 0.17 | 0.25 | 0.48 | -0.02 | 0.45 | -0.23 | -0.05 | 1.00 | -0.39 | 0.49 | -0.45 | -0.40 | -0.02 | -0.46 | 0.38 | -0.06 |
| Positive Affect & Well-Being | 0.61 | -0.65 | -0.19 | -0.32 | -0.79 | 0.26 | -0.73 | 0.46 | 0.27 | -0.39 | 1.00 | -0.61 | 0.81 | 0.68 | 0.27 | 0.79 | -0.56 | 0.26 |
| Psychological Trauma | -0.55 | 0.77 | 0.22 | 0.29 | 0.75 | -0.15 | 0.73 | -0.35 | -0.19 | 0.49 | -0.61 | 1.00 | -0.62 | -0.54 | -0.17 | -0.75 | 0.63 | -0.16 |
| Resilience | 0.62 | -0.68 | -0.18 | -0.30 | -0.78 | 0.22 | -0.72 | 0.45 | 0.26 | -0.45 | 0.81 | -0.62 | 1.00 | 0.66 | 0.24 | 0.76 | -0.59 | 0.25 |
| Satisfaction with SRA | 0.71 | -0.61 | -0.17 | -0.32 | -0.68 | 0.41 | -0.68 | 0.68 | 0.43 | -0.40 | 0.68 | -0.54 | 0.66 | 1.00 | 0.44 | 0.69 | -0.58 | 0.43 |
| Self-Care | 0.38 | -0.18 | -0.04 | -0.18 | -0.25 | 0.88 | -0.28 | 0.73 | 0.84 | -0.02 | 0.27 | -0.17 | 0.24 | 0.44 | 1.00 | 0.26 | -0.29 | 0.87 |
| Self-Esteem | 0.61 | -0.76 | -0.26 | -0.36 | -0.84 | 0.24 | -0.83 | 0.49 | 0.27 | -0.46 | 0.79 | -0.75 | 0.76 | 0.69 | 0.26 | 1.00 | -0.74 | 0.27 |
| Stigma | -0.48 | 0.62 | 0.26 | 0.39 | 0.67 | -0.26 | 0.70 | -0.47 | -0.29 | 0.38 | -0.56 | 0.63 | -0.59 | -0.58 | -0.29 | -0.74 | 1.00 | -0.21 |
| Wheelchair Mobility | 0.39 | -0.13 | 0.04 | -0.12 | -0.23 | 0.82 | -0.25 | 0.70 | 0.85 | -0.06 | 0.26 | -0.16 | 0.25 | 0.43 | 0.87 | 0.27 | -0.21 | 1.00 |

*Note*. Diff. = Difficulty.

**Measurement Invariance: Paraplegia vs. Tetraplegia**

Measurement invariance analyses were conducted to determine whether parameter estimates for Model 8 were invariant across individuals with paraplegia (n=231) and tetraplegia (n=290). The analytic approach for these analyses mirrored those reported in Tulsky *et al*.[21] Briefly, we tested three CFA models with increasing constraints across the subsamples: (1) a configural invariance model (i.e., factor structure equivalence), (1) a metric invariance model (factor loading equivalence), and (3) a scalar invariance model (i.e., indicator intercept equivalence). Worsening of model fit greater than .01 (for CFI and RMSEA) or .015 (for SRMR) was considered indicative of non-invariance.[23] In such cases, we pursued tests of partial measurement invariance using a backward search approach.[24] Using an adjusted p-value of .01, we removed invariance constraints sequentially one at a time based on the largest modification index values until all modification indices were no longer significant.

The configural invariance model fit the data well: χ2 (232) = 454.1, RMSEA = .063 [90% C.I. = .054, .072], CFI = .970, TLI = .960, SRMR = .034, BIC = 18249.9. Likewise, the metric invariance model was supported in terms of global model fit—χ2 (243) = 478.4, RMSEA = .064 [90% C.I. = .055, .073], CFI = .968, TLI = .959, SRMR = .034, BIC = 18207.5—as well as in comparison to the configural invariance model—ΔRMSEA = .001; ΔCFI = .002; ΔSRMR = .009. Inspection of modification indices suggest non-invariance of two factor loadings, however, for Wheelchair Mobility (χ2 = 10.582) and Fine Motor Function (χ2 = 7.418). Furthermore, estimation of an initial partial scalar invariant model (i.e., without loading/intercept constraints for these two indicators) further suggested non-invariance of factor loadings and intercepts for the other two indicators of Physical Function. Therefore, despite acceptable fit compared to the configural invariance model, we re-estimated a partial metric invariance model in which all Physical Function factor loadings were freely estimated across groups: χ2 (240) = 462.6, RMSEA = .062 [90% C.I. = .053, .071], CFI = .970, TLI = .961, SRMR = .039, BIC = 18209.5, ΔRMSEA = -.001; ΔCFI = .000; ΔSRMR = .005, which supported all loading constraints beyond the Physical Function factor. A partial scalar invariance model in which all factor loading and intercept parameters were equated across subgroups except for the Physical Function measures fit the data well and did not result in appreciable fit decrement compared to the partial metric invariance model: χ2 (248) = 487.1, RMSEA = .063 [90% C.I. = .054, .072], CFI = .968, TLI = .960, SRMR = .040, BIC = 18184.2, ΔRMSEA = .001; ΔCFI = .002; ΔSRMR = .001. Despite meeting fit criteria, inspection of modification indices for this model suggested intercept parameters for Bladder Management Difficulties and Bowel Management Difficulties were not invariant across subgroups; however, comparison of factor scores across models in which the intercepts for these indicators were constrained vs. freely estimated suggested negligible differences. Mean bias, the average of factor score differences across models, was .08 (SD = .13) on a standardized metric. Therefore, these results collectively suggest that all factors except Physical Function meet metric and scalar invariance across individuals with paraplegia and tetraplegia.

Supplementary Material References:

1.	Tulsky, D. S., Kisala, P. A., Victorson, D., Choi, S. W., Gershon, R., Heinemann, A. W., & Cella, D. (2015). Methodology for the development and calibration of the SCI-QOL item banks. J Spinal Cord Med, 38(3), 270-287.

2.	Kisala, P. A., & Tulsky, D. S. (2010). Opportunities for CAT applications in medical rehabilitation: development of targeted item banks. J Appl Meas, 11(3), 315-330.

3.	Tulsky, D. S., Kisala, P. A., Victorson, D., Tate, D., Heinemann, A. W., Amtmann, D., & Cella, D. (2011). Developing a contemporary patient-reported outcomes measure for spinal cord injury. Arch Phys Med Rehabil, 92(10 Suppl), S44-51.

4.	Bertisch, H., Kalpakjian, C. Z., Kisala, P. A., & Tulsky, D. S. (2015). Measuring positive affect and well-being after spinal cord injury: Development and psychometric characteristics of the SCI-QOL Positive Affect and Well-being bank and short form. J Spinal Cord Med, 38(3), 356-365.

5.	Tulsky, D. S., Kisala, P. A., Kalpakjian, C. Z., Bombardier, C. H., Pohlig, R. T., Heinemann, A. W., Carle, A., & Choi, S. W. (2015). Measuring depression after spinal cord injury: Development and psychometric characteristics of the SCI-QOL Depression item bank and linkage with PHQ-9. J Spinal Cord Med, 38(3), 335-346.

6.	Kisala, P. A., Tulsky, D. S., Kalpakjian, C. Z., Heinemann, A. W., Pohlig, R. T., Carle, A., & Choi, S. W. (2015). Measuring anxiety after spinal cord injury: Development and psychometric characteristics of the SCI-QOL Anxiety item bank and linkage with GAD-7. J Spinal Cord Med, 38(3), 315-325.

7.	Kisala, P. A., Tulsky, D. S., Pace, N., Victorson, D., Choi, S. W., & Heinemann, A. W. (2015). Measuring stigma after spinal cord injury: Development and psychometric characteristics of the SCI-QOL Stigma item bank and short form. J Spinal Cord Med, 38(3), 386-396.

8.	Victorson, D., Tulsky, D. S., Kisala, P. A., Kalpakjian, C. Z., Weiland, B., & Choi, S. W. (2015). Measuring resilience after spinal cord injury: Development, validation and psychometric characteristics of the SCI-QOL Resilience item bank and short form. J Spinal Cord Med, 38(3), 366-376.

9.	Kalpakjian, C. Z., Tulsky, D. S., Kisala, P. A., & Bombardier, C. H. (2015). Measuring grief and loss after spinal cord injury: Development, validation and psychometric characteristics of the SCI-QOL Grief and Loss item bank and short form. J Spinal Cord Med, 38(3), 347-355.

10.	Kalpakjian, C. Z., Tate, D. G., Kisala, P. A., & Tulsky, D. S. (2015). Measuring self-esteem after spinal cord injury: Development, validation and psychometric characteristics of the SCI-QOL Self-esteem item bank and short form. J Spinal Cord Med, 38(3), 377-385.

11.	Kisala, P. A., Tulsky, D. S., Choi, S. W., & Kirshblum, S. C. (2015). Development and psychometric characteristics of the SCI-QOL Pressure Ulcers scale and short form. J Spinal Cord Med, 38(3), 303-314.

12.	Tulsky, D. S., Kisala, P. A., Tate, D. G., Spungen, A. M., & Kirshblum, S. C. (2015). Development and psychometric characteristics of the SCI-QOL Bladder Management Difficulties and Bowel Management Difficulties item banks and short forms and the SCI-QOL Bladder Complications scale. J Spinal Cord Med, 38(3), 288-302.

13.	Cohen, M. L., Kisala, P. A., Dyson-Hudson, T. A., & Tulsky, D. S. (2017). Measuring pain phenomena after spinal cord injury: Development and psychometric properties of the SCI-QOL Pain Interference and Pain Behavior assessment tools. The Journal of Spinal Cord Medicine, 1-14.

14.	Heinemann, A. W., Kisala, P. A., Hahn, E. A., & Tulsky, D. S. (2015). Development and psychometric characteristics of the SCI-QOL Ability to Participate and Satisfaction with Social Roles and Activities item banks and short forms. J Spinal Cord Med, 38(3), 397-408.

15.	Tulsky, D. S., Kisala, P. A., Victorson, D., Tate, D. G., Heinemann, A. W., Charlifue, S., Kirshblum, S. C., Fyffe, D., Gershon, R., Spungen, A. M., Bombardier, C. H., Dyson-Hudson, T. A., Amtmann, D., C, Z. K., S, W. C., Jette, A. M., Forchheimer, M., & Cella, D. (2015). Overview of the Spinal Cord Injury - Quality of Life (SCI-QOL) measurement system. J Spinal Cord Med, 38(3), 257-269.

16.	Tulsky, D. S., & Kisala, P. A. (2022). Overview of the Spinal Cord Injury-Functional Index (SCI-FI): Structure and Recent Advances. Arch Phys Med Rehabil, 103(2), 185-190.

17.	Tulsky, D. S., Jette, A. M., Kisala, P. A., Kalpakjian, C., Dijkers, M. P., Whiteneck, G., Ni, P., Kirshblum, S., Charlifue, S., Heinemann, A. W., Forchheimer, M., Slavin, M. D., Houlihan, B., Tate, D. G., Dyson-Hudson, T., Fyffe, D. G., Williams, S., & Zanca, J. (2012). Spinal cord injury-functional index: item banks to measure physical functioning in individuals with spinal cord injury. Arch Phys Med Rehabil, 93(10), 1722-1732.

18.	Jette, A. M., Slavin, M. D., Ni, P., Kisala, P. A., Tulsky, D. S., Heinemann, A. W., Charlifue, S., Tate, D. G., Fyffe, D., Morse, L., Marino, R., Smith, I., & Williams, S. (2015). Development and initial evaluation of the SCI-FI/AT. J Spinal Cord Med, 38(3), 409-418.

19.	Slavin, M. D., Kisala, P. A., Jette, A. M., & Tulsky, D. S. (2010). Developing a contemporary functional outcome measure for spinal cord injury research. Spinal Cord, 48(3), 262-267.

20.	PROMIS. (2013). PROMIS Instrument Development and Psychometric Evaluation Scientific Standards. Retrieved 05/24/2022, from <https://www.fda.gov/media/137976/download>

21.	Tulsky, D. S., Boulton, A. J., Kisala, P. A., Tyner, C. E., Pohlig, R., Chiaravalloti, N. D., Slotkin, J., Taylor, H. B., & Sherer, M. (2026). Using confirmatory factor analysis to identify symptom clusters in individuals with acquired disabilities. Qual Life Res, 35(5).

22.	Sherer, M., Poritz, J. M. P., Tulsky, D., Kisala, P., Leon-Novelo, L., & Ngan, E. (2020). Conceptual Structure of Health-Related Quality of Life for Persons With Traumatic Brain Injury: Confirmatory Factor Analysis of the TBI-QOL. Arch Phys Med Rehabil, 101(1), 62-71.

23.	Chen, F. F. (2007). Sensitivity of goodness of fit indexes to lack of measurement invariance. Struct Equ Modeling, 14(3), 464-504.

24.	Jung, E., & Yoon, M. (2016). Comparisons of three empirical methods for partial factorial invariance: Forward, backward, and factor-ratio tests. Struct Equ Modeling, 23(4), 567-584.
